# Genome-scale copy number variant analysis in schizophrenia patients and controls from South India

**DOI:** 10.3389/fnmol.2023.1268827

**Published:** 2023-12-21

**Authors:** Minali Singh, Dibyabhabha Pradhan, Poornima Kkani, Gundugurti Prasad Rao, Naveen Kumar Dhagudu, Lov Kumar, Chellamuthu Ramasubramanian, Srinivasan Ganesh Kumar, Surekha Sonttineni, Kommu Naga Mohan

**Affiliations:** ^1^Molecular Biology and Genetics Laboratory, Department of Biological Sciences, Birla Institute of Technology and Science, Pilani – Hyderabad Campus, Hyderabad, India; ^2^Centralized Core Research Facility, All India Institute of Medical Sciences, New Delhi, India; ^3^Department of Zoology, Thiagarajar College, Madurai, India; ^4^Asha Hospital Institute of Medical Psychology, Hyderabad, India; ^5^ESIC Medical College and Hospital, Hyderabad, India; ^6^Department of Computer Engineering, National Institute of Technology, Kurukshetra, India; ^7^Department of Psychiatry, M.S. Chellamuthu Trust and Research Foundation, Madurai, India; ^8^Centre for Human Disease Research, Birla Institute of Technology and Science, Pilani – Hyderabad Campus, Hyderabad, India

**Keywords:** schizophrenia, India, CNVs, case–control studies, deletions, duplications, PsychArray

## Abstract

Copy number variants (CNVs) are among the main genetic factors identified in schizophrenia (SZ) through genome-scale studies conducted mostly in Caucasian populations. However, to date, there have been no genome-scale CNV reports on patients from India. To address this shortcoming, we generated, for the first time, genome-scale CNV data for 168 SZ patients and 168 controls from South India. In total, 63 different CNVs were identified in 56 patients and 46 controls with a significantly higher proportion of medium-sized deletions (100 kb–1 Mb) after multiple testing (FDR = 2.7E-4) in patients. Of these, 13 CNVs were previously reported; however, when searched against GWAS, transcriptome, exome, and DNA methylation studies, another 17 CNVs with candidate genes were identified. Of the total 30 CNVs, 28 were present in 38 patients and 12 in 27 controls, indicating a significantly higher representation in the former (*p* = 1.87E-5). Only 4q35.1-q35.2 duplications were significant (*p* = 0.020) and observed in 11 controls and 2 patients. Among the others that are not significant, a few examples of patient-specific and previously reported CNVs include deletions of 11q14.1 (*DLG2*), 22q11.21, and 14q21.1 (*LRFN5*). 16p13.3 deletion (*RBFOX1*), 3p14.2 duplication (*CADPS*), and 7p11.2 duplication (*CCT6A*) were some of the novel CNVs containing candidate genes. However, these observations need to be replicated in a larger sample size. In conclusion, this report constitutes an important foundation for future CNV studies in a relatively unexplored population. In addition, the data indicate that there are advantages in using an integrated approach for better identification of candidate CNVs for SZ and other mental health disorders.

## Introduction

Schizophrenia is a complex neuropsychiatric disorder with symptoms such as delusions, hallucinations, cognitive impairment, and lack of social interest. Although the exact cause is not established, both genetic and environmental factors are well recognized as playing a role in its occurrence (Wahbeh and Avramopoulos, 2021). In the case of genetic evidence, there is a 6-fold risk in families with an affected first-degree relative, whereas in monozygotic and dizygotic twins, the risk increases by 48- and 17-fold, respectively (Gottesman, 1994).

Multiple large-scale genome-wide association studies (GWAS) involving cases and controls identified three kinds of risk variants: single nucleotide polymorphisms (SNPs), copy number variants (CNVs), and *de novo* mutations (DNMs) (Kirov et al., 2012; Rees et al., 2014; Ripke et al., 2014; Marshall et al., 2017). Of these, CNVs involve segmental deletion or duplication of a DNA segment ranging in size from 1 kb to several Mb, resulting in a dosage imbalance of genes equivalent to haplo-insufficiency in cases that involve deletions and triplosenstivity in cases of duplications (Rees and Kirov, 2021).

To date, most genome-scale CNV studies have been conducted in Caucasian populations and a few in Asian populations but none have been undertaken for populations in India (Ikeda et al., 2010; Rees et al., 2014; Li et al., 2016; Kushima et al., 2017; Marshall et al., 2017). Since India is the most populated country in the world, this lack of genome-scale studies impedes understanding of the contribution of CNVs to SZ in this region. In this study, we first identified CNVs in 168 SZ patients and 168 age- and sex-matched controls from South India using PsychArrays. The CNVs identified in the sample set were searched against databases that contain previously reported CNVs. To detect unreported CNVs with candidate genes, GWAS, *de novo* mutation, methylation, and expression data from the SZDB V2.0 were used (Wu et al., 2017).

## Materials and methods

### Generation of genotyping data, quality control filtering, and relatedness testing

A total of 384 samples containing 194 schizophrenia cases (mean age ± SD: 36.4 ± 12.4; women = 48%) and 190 age-and sex-matched controls (mean age ± SD: 36.7 ± 10.2; women = 46%) of South Indian origin were identified based on the Diagnostic Statistic Manual-5 (DSM-V) criteria by an experienced psychiatrist. Written consent for peripheral blood sampling was obtained from all the participants or their legally authorized relatives. Genomic DNAs from the blood samples were genotyped using Illumina’s Infinum^™^ PsychArray v 1.3 (Illumina, San Diego, California, USA) at Sandor Life Sciences (Hyderabad, India). The intensity data obtained were used for sample and SNP-level quality control. GenomeStudio was used for data pre-processing using the clustering algorithm with a quality cutoff score of 0.15 (Bacchelli et al., 2020), and samples with low GenCall (GC) scores, having call rates below 0.98, and those of unknown sex were excluded from the analysis. These QC-passed data were subjected to further quality control using PLINK1.9 (Purcell et al., 2007), and SNPs with >5% missing call rates, those with a minor allele frequency of less than 1%, and those that failed the Hardy–Weinberg equilibrium (HWE; case value of *p* < 1 × 10^−6^, control value of *p*: <1 × 10^−10^) were removed from analyses (Rodríguez-López et al., 2018; Behera et al., 2023). Samples with 5% missing calls, sex discrepancy, and those deviating by three standard deviations from the heterozygosity rate were then excluded. Cryptic relatedness between individuals was detected based on pairwise identity-by-descent analysis, wherein any member of each pair of individuals with PIHAT >0.2 was removed (Marees et al., 2018). Using the remaining samples, MDS-based clustering was performed to study the population structure using PLINK1.9, and data were plotted using R-program to verify an appropriate overlap of the cases and controls and, to identify any outliers.

### CNV calling

CNV calling was performed using the PennCNV algorithm to identify regions with copy number variations following standard pipelines that mainly focus on autosomes (Wang et al., 2007; Fang and Wang, 2018). First, the intensity files were used to generate PennCNV-compatible log R ratio (LRR) and B-allele frequency (BAF) input files for each sample. Then, a custom population B-allele frequency file (PFB) was generated using the “compile_pfb.pl” script followed by a GC model signal file specific for PsychArray using “cal_gc_snp.pl” to adjust the genomic waves and reduce false-positive CNV calls. CNVs were detected using the “detect_cnv.pl” script, and the adjacent calls were merged using “clean_cnv.pl” if two successive CNVs provided a gap of less than 20% of the total length. For CNV filtering based on the sample level, individuals with a standard deviation of log R ratio ≥ 0.3 were excluded. Furthermore, CNVs based on call-level filtration (≥10 kb with ≥30 consecutive probes) were identified using the “filter_cnv.pl” script (Vega-Sevey et al., 2020). The centromeric and telomeric regions were compiled from UCSC (hg19 assembly) and CNV calls overlapping with ≥50% of these chromosomal regions were removed by running the “scan_region.pl” script (Karolchik et al., 2004; Fang and Wang, 2018) using PennCNV. For annotating the identified CNVs, we used the hg19 reference gene annotation file from the PennCNV package using script “scan_region.pl.” The coordinates of the shortlisted CNVs were viewed in the UCSC browser to determine cytogenetic band information. CNVs were classified as non-recurrent or recurrent based on their occurrence in one or more individuals, respectively.

### Statistical analyses

Significant differences between cases and controls were identified using the two-tailed Fisher’s exact test.^1^ Odds ratios were calculated using MedCalc Software Ltd. version 22.007.^2^ For multiple testing of the association of CNVs based on size, we used the Benjamini–Hochberg method of false discovery rate (FDR). Briefly, the *p*-values obtained by the Fisher’s test were ranked, and FDRs were calculated based on the formula: FDR = $\frac{n\;x\;p-\text{value}\;}{\text{Rank}\;\text{number}}$, where *n* = number of different CNV types tested (see Table 1; Thissen et al., 2002). Post-hoc statistical power was calculated using the frequencies of CNVs observed in patients and controls, the sample sizes used, and the type I error rate of 0.05 using the ClinCalc tool.^3^

**Table 1:** Number of different sized deletions and duplications identified in controls and patients.

CNV type	Total unique identified	Controls		Patients		*p*-value	FDR
		Present	Absent	Present	Absent		
Large-sized deletions	3	0	3	3	0	0.1000	NS
Medium-sized deletions	20	8	12	20	0	4.5E-5	2.7E-4
Small-sized deletions	1	1	0	1	0	1.0000	NS
Large-sized duplications	2	1	1	1	1	1.0000	NS
Medium-sized duplications	36	36	0	30	6	0.0249	0.0747
Small-sized duplications	1	0	1	1	0	1.0000	NS

Gray row indicates CNVs showing significant difference between controls and patients. NS, Not significant; FDR, False discovery rate calculated by Benjamini-Hochberg method.

### Identification of CNVs with potential association with SZ

We first filtered CNVs as reported or unreported based on their presence and association with SZ in the SZDB2.0 database. Unreported CNVs were further queried against the genes identified by GWAS (11,260 patients and 24,542 controls), exome sequencing (14,598 patients and 11,515 controls), and DNA methylation analyses (191 patients and 335 controls) reported in the same database. Genome-wide transcriptome studies reported by the CommonMind and PsychEncode Consortia (817 patients and 1,115 controls) were used to identify a subset of the CNVs containing the candidate genes (Gandal et al., 2018; Hoffman et al., 2019). CNVs that were not present in the SZDB2.0 and that did not contain the candidate genes were considered to not be associated with SZ. Wherever possible, the frequencies of the observed CNVs in the present population were compared with those reported for other populations.

### Calculation of penetrance

The number of controls and patients containing 22q11.2 deletions and the total number of samples tested were obtained from the SZDB 2.0. Using an incidence of 0.7%, penetrance and its critical intervals were calculated using CalPen software (Addepalli et al., 2020).

## Results

**Figure 1:**
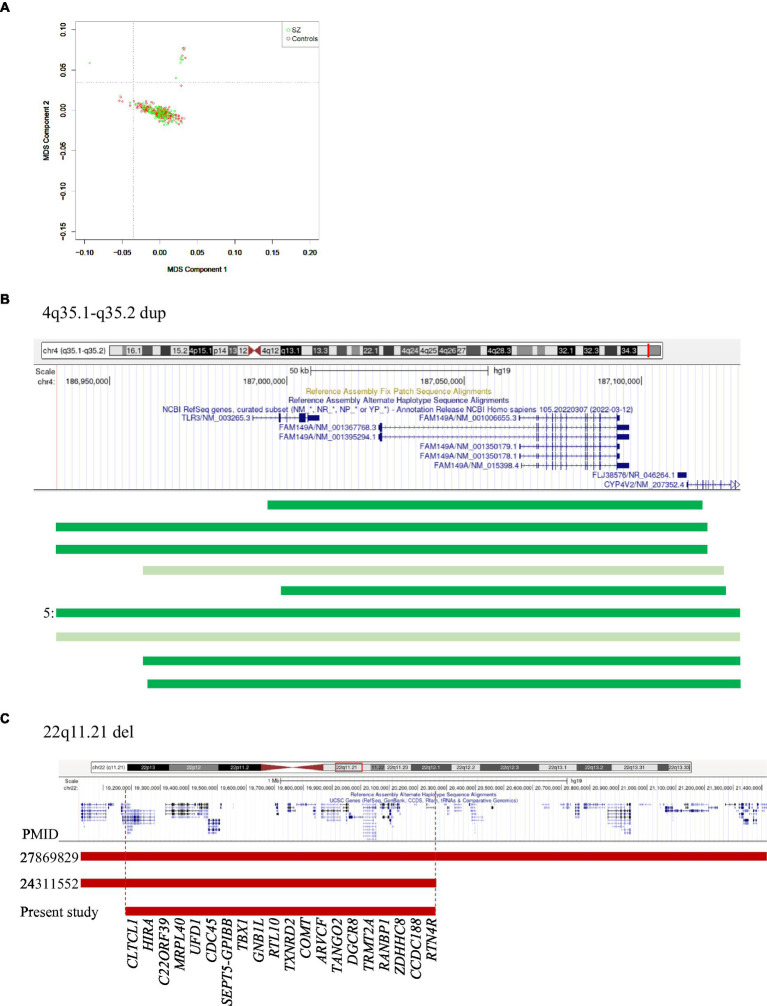
**(A)** MDS plot of controls and patients. **(B)** 4q35.1-q35.2 region showing duplications in 11 controls (dark green) and 2 patients (light green). 5 indicates that five controls had identical duplications. **(C)** 22q11.21 region showing reported and observed deletions. Vertical dotted lines indicate the minimal region. The numbers on the left indicate PMIDs corresponding to Marshall et al. (2017; 27869829) and Rees et al. (2014; 24311552).

Of the 194 patients and 190 controls used, 373 samples passed filtering for low GenCall (GC) scores, call rates <0.98, and unknown sex. A further 11 samples were excluded due to ambiguous sex calls. SNPs that did not conform to HWE or showed >5% missing call rates or MAF < 1.0% were removed from further analysis. This resulted in 273,175 SNPs with which heterozygosity analysis was used to eliminate one outlier, resulting in 361 samples. Cryptic relatedness testing using PIHAT further identified another 13 patients and 12 controls with scores ≥0.2 that were removed to finally obtain 336 analyzable samples (168 controls and 168 patients). MDS clustering to study population stratification confirmed an appropriate overlap of the controls and patients with no outliers and were suitable for CNV detection (Figure 1A).

All the 336 samples had standard deviations of log R ratios <0.3 and gave an initial list of 3,110 CNVs which when filtered based on size (≥10 kb), the presence of ≥30 consecutive probes gave 122 variants, of which 106 were genic. Four of them were further removed because of their overlaps with centromeric and telomeric regions. Of the remaining 102 CNVs, 39 were represented more than once, leaving 63 unique CNVs. The size distribution of these 63 CNVs is summarized in Table 1. The medium-sized CNVs (0.1–1.0 Mb) were higher in patients than in controls (*p* = 0.1975), but the difference was significant in case of deletions (20 vs. 8; *p* = 4.5E-5; FDR = 2.7E-4). Of the 63 different CNVs, 45 were present in patients and 30 in controls, including 12 that were shared between the two groups (Supplementary Table 1). These data suggest ~1.5-fold representation in patients (*p* = 0.0107) over controls.

In total, 13 of the 63 different CNVs observed in 17 patients and 7 controls were previously reported (Table 2). Of these, seven were present only in patients and two in only controls, whereas the remaining four were shared. When the remaining 50 unreported CNVs were tested for the presence of candidate genes (mentioned in the section Materials and Methods), an additional 17 were identified (Table 2). Of these, 11 CNVs were present exclusively in patients, and the remaining 6 were shared with controls. In total, of the 30 different CNVs (17 unreported with candidate genes and 13 reported), 28 were present in patients and 12 in controls, indicating a significant difference in their representation (*p* = 1.87E-5). This difference was mainly because of a higher number of medium-sized deletions in patients as mentioned above.

**Table 2:** List of reported and unreported CNVs with candidate genes identified in the patients and controls in the present study and their frequencies in other populations.

Reported CNVs											
CNV (coordinate)	Present study				Published reports						
	No. of patients	No. of controls	Odds ratio (CI interval)	*p*-value	Patients		Controls		Odds ratio (CI interval)	*p*-value	
					With CNV	Total	With CNV	Total			Study group
6p21.33 del (chr6:31360255–31453618)	1	1	1.00 (0.06–16.1)	1.00	6	8,290	4	7,431	1.34 (0.38–4.77)	0.64	American
8p23.2 del (chr8:3681516–3832465)	2	1	2.01 (0.18–22.4)	0.57	6	8,290	2	7,431	2.69 (0.54–13.3)	0.2256	American
11q14.1 del (chr11:83930211–84642163)	1	0	3.01 (0.12–74.6)	0.49	3	1,699	0	824	3.40 (0.17–65.9)	0.42	Chinese and Japanese
14q21.1 del (chr14:41855338–43171423)	1	0	3.01 (0.12–74.6)	0.49	NA	NA	NA	NA	NA	NA	Family studies
15q11.2 del (chr15:22755185–23236972)	2	0	5.06 (0.24–106.2)	0.29	44	6,882	47	11,255	1.53 (1.01–2.32)	0.04	Multiple populations
					6	307	3	359	2.37 (0.59–9.53)	0.23	South Indian
20p12.1 del (chr20:14890211–15122254)	1	0	3.01 (0.12–74.6)	0.49	1	454	0	416	2.75 (0.11–67.67)	0.54	Canadian
22q11.21 del (chr22:19195680–20267213)	1	0	3.01 (0.12–74.6)	0.49	64	21,094	1	20,227	88.9 (12.34–641)	<0.0001	European, Asian, African American
4q13.2-q13.3 dup (chr4:69697128–71389594)	1	0	3.01 (0.12–74.6)	0.49	1	3,518	0	4,328	3.61 (0.15–88.7)	0.43	European
7q36.2 dup (chr7:153154107–153652188)	1	0	3.01 (0.12–74.6)	0.49	7	8,290	3	7,431	2.09 (0.54–8.1)	0.28	American
7q21.11 dup (chr7:83179622–83437224)	0	1	0.33 (0.01–8.19)	0.49	1	1,699	0	824	1.46 (0.06–35.79)	0.81	Chinese and Japanese
15q11.2 dup (chr15:22755185–23236972)	1	1	1.00 (0.06–16.1)	1.00	23	8,290	16	7,431	1.29 (0.68–2.44)	0.43	American
16p13.11 dup (chr16:15548310–16291983)	0	1	0.33 (0.01–8.19)	0.49	37	12,029	93	69,289	2.3 (1.57–3.36)	<0.0001	Multiple populations
22q11.22 dup (chr22:22316631–22555078)	5	2	2.55 (0.48–13.3)	0.26	NA	NA	NA	NA	NA	NA	Family studies

Unreported CNVs with candidate genes								
CNVs with Odds ratio-3.01; CI-0.12-74.61 and *p* value 0.49								
CNV	Coordinate	No. of patients	No. of controls	Candidate gene	GWAS	Exome	Transcriptome and eQTL	Differential methylation and meQTL
2q32.1-q32.2 del	(chr2:186067813–190115961)	1	0	CALCRL	***	NA	***	***
				COL3A1	*	PD	***	***
				DIRC1	***	PD	NA	NA
				ITGAV	*	D	***	***
				ZSWIM2	*	PD	***	***
11p15.3 del	(chr11:10970735–11294339)	1	0	GALNT18	NA	NA	***	***
1q42.13 dup	(chr1:228467711–228564884)	1	0	OBSCN	–	PD	***	***
2p22.3 Dup	(chr2:32713706–33152042)	1	0	BIRC6	**	PD	***	***
7p11.2 Dup	(chr7:55804513–56294281)	1	0	CCT6A	*	B	NS	***
7q21.3 Dup	(chr7:96567080–97107457)	1	0	DLX5	***	NA	***	***
9p22.1 Dup	(chr9:18882562–19192694)	1	0	ADAMTSL1	***	B	***	***
13q12.3-q13.1 Dup	(chr13:32175095–32525678)	1	0	EEF1DP3	*	NA	NS	***
CNVs with Odds ratio-5.06; CI-0.24-106.20 and *p* value 0.29								
16p13.3 del	(chr16:538565–863498)	2	0	CAPN15		PD	***	***
3p14.2 Dup	(chr3:62611243–63258941)	2	0	CADPS	***	NA	NS	***
CNVs with Odds ratio-1.00; CI-0.06-16.12 and *p* value 1.00								
16p13.3 del	(chr16:6837553–6960281)	1	1	RBFOX1	***	NA	***	***
11p15.4 Dup	(chr11:9645397–10143942)	1	1	SBF2	***	NA	***	***
11q22.3 Dup	(chr11:106956255–107426129)	1	1	CWF19L2	***	PD	***	***
16p13.3 Dup	(chr16:6546624–6672741)	1	1	Same as in deletion				
CNVs with Odds ratio-0.17; CI-0.03-0.78 and *p* value 0.02								
4q35.1-q35.2 Dup	(chr4:186935500–187122319)	2	11	FAM149A	***	NA	***	***
				TLR3	*	B	NS	***
CNVs with Odds ratio-0.24; CI-0.02-2.22 and *p* value 0.21								
7q11.23 Dup	(chr7: 76131646–76637441)	1	4	DTX2	**	PD	***	***
				POMZP3	**	D	***	***
CNVs with Odds ratio-1.50; CI-0.24-9.14 and *p* value 0.65								
19p13.3 Dup	(chr19:789890–1397443)	3	2	Same as in deletion				

Unreported CNVs do not have population-level data. NA, No data available; NS, Not significant; D, Damaging mutation; PD, Probably damaging mutation; B, Benign; ‘–’ *p* value close to 0.05, *p value 0.05–0.02, **p value 0.02–0.01, ***p value <0.001.

### 4q35.1-q35.2 duplication

This was the most common CNV in our dataset, measured ~0.19 Mb containing *CYP4V2, FAM149A, FLJ38576*, and *TLR3* genes, and occurred at a significantly higher number in controls than patients (11 versus 2, respectively; *p* = 0.02). Two of the controls had partial duplications of *TLR3* (Figure 1B), a member of the highly conserved toll-like receptors that play a role in innate immunity. Experimental data suggest that *TLR3* negatively controls the expression of *DISC1*, resulting in impaired dendritic arborization. CNVs of this region have not been reported, but there was a nominal association of an intronic variant (*rs3775294*) in SZ (*p* = 0.046) (Pardiñas et al., 2018). Both *TLR3* and *FAM149A* were implicated in GWAS, expression, and methylation studies on SZ patients (Wu et al., 2017).

None of the other CNVs have a significant difference in their occurrence between the patients and controls studied. A few that have been previously reported and the unreported ones with candidate genes are mentioned here: (i) Duplications of 22q11.22 (~0.24 Mb) involving *PRAMENP* and the first 13 exons of *TOP3β* were observed in five patients and two controls. This was the second most common CNV in this dataset. (ii) 15q11.2 deletions were found in two patients but not in controls, whereas duplications were present in one patient and one control. When combined with the data from Saxena et al. (2019), both deletions and duplications were not significantly different in a total of 540 controls and 499 patients. CNVs of this region were considered as variants of uncertain significance (VUS; Mohan et al., 2019). (iii) 11q14.1 deletion (~0.71 Mb) involving *DLG2* was found in one patient. Deletions of this region were reported in autism spectrum disorders (Griesius et al., 2022) and SZ patients in Caucasian and Asian populations (Walsh et al., 2008; Kushima et al., 2017). (iv) 14q21.1 deletion (~1.3 Mb) involving *LRFN5* was found in one patient and was previously reported in SZ, developmental delay, intellectual disability, and microcephaly (Xu et al., 2009; Lybaek et al., 2022). The observed deletion was larger, involving the entire gene as well as the 60 kb region implicated in its regulation with conformation differences between maternally and paternally transmitted chromosomes (Mikhail et al., 2011). (v) 22q11.21 deletion (~1.1 Mb) was observed in one patient and is smaller than the previously reported ones (~2.35 Mb and ~ 1.24 Mb; Rees et al., 2014; Marshall et al., 2017). The observed deletion did not include *DGCR2, ESS2, TSSK2, GSC2, SLC25A1*, and *CLTCL1* (Figure 1C). Deletions of this region are considered to have higher penetrance for SZ (Addepalli et al., 2020). For example, the data given in Table 2 gave a penetrance value of 0.272 with critical intervals of 0.079 to 0.818. (vi) 3p14.2 duplations of ~0.65 Mb affecting the copy number of *CADPS* were observed in two patients. Duplications of this gene are not reported in the literature, but duplication of *CADPS2*, a member of the same gene family, was reported in autism spectrum disorders (Girirajan et al., 2013).

### Statistical power considerations

As mentioned above, of the total of 30 CNVs identified, 28 were present in 38 patients (22.6%), whereas 12 were present in 27 controls (16.1%). These incidences, along with a Type I error rate of 0.05, the data on 168 patients and 168 controls yielded a post-hoc statistical power of 32.5%.

## Discussion

The present study is the first to describe. CNVs in Indian SZ patients and controls, and compare the data with other ethnic groups. The data were in overall agreement with the increased CNV burden observed in SZ in other populations (Marshall et al., 2017). Specifically, there was a significant enrichment of medium-sized deletions that have been previously reported or those containing SZ-associated genes in the patients studied here. These data also suggest the utility of PsychArrays in cost-effectively validating previous findings and identifying novel CNVs in patients from India and such unexplored ethnic groups. It may be noted that this report on CNVs used 168 patients and 168 controls and thus had a relatively small sample size. In this context, 4q35.1-q35.2 duplication, which was the only CNV with a significantly higher occurrence in controls, requires replication on a larger sample. However, such sample sizes were also used in other similar initial studies (e.g., Vega-Sevey et al., 2020; Abumadini et al., 2023).

In this initial search, only 13 out of the 63 different identified CNVs were previously reported in SZ. However, we reasoned that more relevant CNVs could be found if genes identified through GWAS, methylation, exome, and transcriptome studies were also considered. This approach draws support from a previous study on German and Chinese patients, wherein the possibility of the occurrence of CNVs was successfully tested based on *GABRB2* variants reported in other studies (Ullah et al., 2020). In agreement with these expectations, 17 additional CNVs were identified in our dataset. Thus, an integrated approach of a similar kind is worthwhile implementing for identifying unreported CNVs that are of potential interest from publicly available data and for confirmation through replication studies for SZ and other mental health disorders. As mentioned in the ‘Results’ section, a few CNVs, such as 3p14.2 duplication, occurred only in patients but not in controls. This occurrence is at present not significant, but replication studies are needed to confirm any association between the reported and unreported CNVs described here.

A general observation among case–control studies on other populations suggests that the frequencies of many of the CNVs involved are less than 1% (Rees et al., 2014; Marshall et al., 2017). For instance, 15q11.2 del, which is the most common CNV in SZDB2.0, has a frequency of 0.46%, whereas 16p11.2 duplications that were highest in the patients studied by Marshall et al. (2017), showed a frequency of 0.33%. It is noteworthy that a majority of the SZ-associated CNVs that were identified in this study and listed in SZDB2.0 also do not have significant *p*-values except for 15q11.2 del, 22q11.21 del, and 16p13.11 dup (*p* = 0.04, <0.0001 and < 0.0001, respectively). Thus, the data suggest that, in general, CNVs with candidate genes are expected to be less frequent. In this context, case–control studies with access to samples from family members (affected as well as unaffected) will prove valuable in determining the contribution of these CNVs to SZ.

## Data availability statement

The data presented in the study are deposited in the NCBI GEO repository with accession number GSE242813.

## Ethics statement

The studies involving humans were approved by Institutional Human Ethics Committees of Birla Institute of Technology and Science, Pilani. Hyderabad, and all collaborating institutions. The studies were conducted in accordance with the local legislation and institutional requirements. The participants provided their written informed consent to participate in this study.

## Author contributions

MS: Writing – review & editing, Data curation, Formal analysis, Investigation, Methodology, Software, Validation, Writing – original draft. DP: Writing – review & editing, Data curation, Methodology, Formal analysis, Investigation, Software. PK: Investigation, Writing – review & editing. GP: Investigation, Writing – review & editing. ND: Investigation, Writing – review & editing. LK: Investigation, Writing – review & editing, Software. CR: Investigation, Writing – review & editing. SK: Investigation, Writing – review & editing. SS: Investigation, Writing – review & editing. KM: Investigation, Writing – review & editing, Conceptualization, Funding acquisition, Project administration, Resources, Supervision, Methodology.

## Footnotes

^1^ Graphpad.com

^2^ https://www.medcalc.org/calc/odds_ratio.php

^3^ https://clincalc.com/stats/power.aspx

## References

[ref1] Abumadini M. S. Al Ghamdi K. S. Alqahtani A. H. Almedallah D. K. Callans L. Jarad J. A.. (2023). Genome-wide copy number variant screening of Saudi schizophrenia patients reveals larger deletions in cases versus controls. Front. Mol. Neurosci. 16:1069375. doi: 10.3389/fnmol.2023.1069375 36846569 PMC9950097

[ref2] Addepalli A. Kalyani S. Singh M. Bandyopadhyay D. Mohan K. N. (2020). CalPen (calculator of penetrance), a web-based tool to estimate penetrance in complex genetic disorders. PLoS One 15:e0228156. doi: 10.1371/journal.pone.0228156, PMID: 31995602 PMC6988981

[ref3] Bacchelli E. Cameli C. Viggiano M. Igliozzi R. Mancini A. Tancredi R.. (2020). An integrated analysis of rare CNV and exome variation in autism Spectrum disorder using the Infinium PsychArray. Sci. Rep. 10:3198. doi: 10.1038/s41598-020-59922-3, PMID: 32081867 PMC7035424

[ref4] Behera C. Kaushik R. Bharti D. R. Nayak B. Bhardwaj D. N. Pradhan D.. (2023). PsychArray-based genome wide association study of suicidal deaths in India. Brain Sci. 13:136. doi: 10.3390/brainsci13010136 36672117 PMC9856809

[ref5] Fang L. Wang K. (2018). Identification of copy number variants from SNP arrays using PennCNV. Methods Mol. Biol. 1833, 1–28. doi: 10.1007/978-1-4939-8666-8_1, PMID: 30039360

[ref6] Gandal M. J. Zhang P. Hadjimichael E. Walker R. L. Chen C. Liu S.. (2018). Transcriptome-wide isoform-level dysregulation in ASD, schizophrenia, and bipolar disorder. Science 362:eaat8127. doi: 10.1126/science.aat8127, PMID: 30545856 PMC6443102

[ref7] Girirajan S. Dennis M. Y. Baker C. Malig M. Coe B. P. Campbell C. D.. (2013). Refinement and discovery of new hotspots of copy-number variation associated with autism spectrum disorder. Am. J. Hum. Genet. 92, 221–237. doi: 10.1016/j.ajhg.2012.12.016, PMID: 23375656 PMC3567267

[ref8] Gottesman I. I. (1994). Complications to the complex inheritance of schizophrenia. Clin. Genet. 46, 116–123. doi: 10.1111/j.1399-0004.1994.tb04213.x 7988068

[ref9] Griesius S. O’Donnell C. Waldron S. Thomas K. L. Dwyer D. M. Wilkinson L. S.. (2022). Reduced expression of the psychiatric risk gene DLG2 (PSD93) impairs hippocampal synaptic integration and plasticity. Neuropsychopharmacology 47, 1367–1378. doi: 10.1038/s41386-022-01277-6 35115661 PMC9117295

[ref10] Hoffman G. E. Bendl J. Voloudakis G. Montgomery K. S. Sloofman L. Wang Y.-C.. (2019). CommonMind consortium provides transcriptomic and epigenomic data for schizophrenia and bipolar disorder. Sci. Data 6:180. doi: 10.1038/s41597-019-0183-6, PMID: 31551426 PMC6760149

[ref11] Ikeda M. Aleksic B. Kirov G. Kinoshita Y. Yamanouchi Y. Kitajima T.. (2010). Copy number variation in schizophrenia in the Japanese population. Biol. Psychiatry 67, 283–286. doi: 10.1016/j.biopsych.2009.08.034, PMID: 19880096

[ref12] Karolchik D. Hinrichs A. S. Furey T. S. Roskin K. M. Sugnet C. W. Haussler D.. (2004). The UCSC table browser data retrieval tool. Nucleic Acids Res. 32, 493D–4496D. doi: 10.1093/nar/gkh103 PMC308837 14681465

[ref14] Kirov G. Pocklington A. J. Holmans P. Ivanov D. Ikeda M. Ruderfer D.. (2012). De novo CNV analysis implicates specific abnormalities of postsynaptic signalling complexes in the pathogenesis of schizophrenia. Mol. Psychiatry 17, 142–153. doi: 10.1038/mp.2011.154, PMID: 22083728 PMC3603134

[ref15] Kushima I. Aleksic B. Nakatochi M. Shimamura T. Shiino T. Yoshimi A.. (2017). High-resolution copy number variation analysis of schizophrenia in Japan. Mol. Psychiatry 22, 430–440. doi: 10.1038/mp.2016.88, PMID: 27240532

[ref16] Li Z. Chen J. Xu Y. Yi Q. Ji W. Wang P.. (2016). Genome-wide analysis of the role of copy number variation in schizophrenia risk in Chinese. Biol. Psychiatry 80, 331–337. doi: 10.1016/j.biopsych.2015.11.012 26795442

[ref17] Lybaek H. Robson M. de Leeuw N. Hehir-Kwa J. Y. Jeffries A. Haukanes B. I.. (2022). LRFN5 locus structure is associated with autism and influenced by the sex of the individual and locus conversions. Autism Res. 15, 421–433. doi: 10.1002/aur.2677, PMID: 35088940 PMC9305582

[ref18] Marees A. T. de Kluiver H. Stringer S. Vorspan F. Curis E. Marie-Claire C.. (2018). A tutorial on conducting genome-wide association studies: quality control and statistical analysis. Int. J. Methods Psychiatr. Res. 27:e1608. doi: 10.1002/mpr.1608, PMID: 29484742 PMC6001694

[ref19] Marshall C. R. Howrigan D. P. Merico D. Thiruvahindrapuram B. Wu W. Greer D. S.. (2017). Contribution of copy number variants to schizophrenia from a genome-wide study of 41,321 subjects. Nat. Genet. 49, 27–35. doi: 10.1038/ng.3725, PMID: 27869829 PMC5737772

[ref20] Mikhail F. M. Lose E. J. Robin N. H. Descartes M. D. Rutledge K. D. Rutledge S. L.. (2011). Clinically relevant single gene or intragenic deletions encompassing critical neurodevelopmental genes in patients with developmental delay, mental retardation, and/or autism spectrum disorders. Am. J. Med. Genet. A 155, 2386–2396. doi: 10.1002/ajmg.a.34177 22031302

[ref21] Mohan K. N. Cao Y. Pham J. Cheung S. W. Hoffner L. Ou Z. Z.. (2019). Phenotypic association of 15q11.2 CNVs of the region of breakpoints 1-2 (BP1-BP2) in a large cohort of samples referred for genetic diagnosis. J. Hum. Genet. 64, 253–255. doi: 10.1038/s10038-018-0543-7, PMID: 30542208

[ref22] Pardiñas A. F. Holmans P. Pocklington A. J. Escott-Price V. Ripke S. Carrera N.. (2018). Common schizophrenia alleles are enriched in mutation-intolerant genes and in regions under strong background selection. Nat. Genet. 50, 381–389. doi: 10.1038/s41588-018-0059-2, PMID: 29483656 PMC5918692

[ref23] Purcell S. Neale B. Todd-Brown K. Thomas L. Ferreira M. A. R. Bender D.. (2007). PLINK: a tool set for whole-genome association and population-based linkage analyses. Am. J. Hum. Genet. 81, 559–575. doi: 10.1086/519795, PMID: 17701901 PMC1950838

[ref24] Rees E. Kirov G. (2021). Copy number variation and neuropsychiatric illness. Curr. Opin. Genet. Dev. 68, 57–63. doi: 10.1016/j.gde.2021.02.014, PMID: 33752146 PMC8219524

[ref25] Rees E. Walters J. T. R. Georgieva L. Isles A. R. Chambert K. D. Richards A. L.. (2014). Analysis of copy number variations at 15 schizophrenia-associated loci. Br. J. Psychiatry 204, 108–114. doi: 10.1192/bjp.bp.113.131052, PMID: 24311552 PMC3909838

[ref26] Ripke S. Neale B. M. Corvin A. Walters J. T. R. Farh K.-H. Holmans P. A.. (2014). Biological insights from 108 schizophrenia-associated genetic loci. Nature 511, 421–427. doi: 10.1038/nature13595 25056061 PMC4112379

[ref27] Rodríguez-López J. Flórez G. Blanco V. Pereiro C. Fernández J. M. Fariñas E.. (2018). Genome wide analysis of rare copy number variations in alcohol abuse or dependence. J. Psychiatr. Res. 103, 212–218. doi: 10.1016/j.jpsychires.2018.06.001 29890507

[ref28] Saxena S. Kkani P. Ramasubramanian C. Kumar S. G. Monisha R. Prasad Rao G.. (2019). Analysis of 15q11.2 CNVs in an Indian population with schizophrenia. Ann. Hum. Genet. 83, 187–191. doi: 10.1111/ahg.12300, PMID: 30779116

[ref29] Thissen D. Steinberg L. Kuang D. (2002). Quick and easy implementation of the Benjamini-Hochberg procedure for controlling the false positive rate in multiple comparisons. J. Educ. Behav. Stat. 27, 77–83. doi: 10.3102/10769986027001077

[ref30] Ullah A. Long X. Mat W.-K. Hu T. Khan M. I. Hui L.. (2020). Highly recurrent copy number variations in GABRB2 associated with schizophrenia and premenstrual dysphoric disorder. Front. Psych. 11:572. doi: 10.3389/fpsyt.2020.00572, PMID: 32695026 PMC7338560

[ref31] Vega-Sevey J. G. Martínez-Magaña J. J. Genis-Mendoza A. D. Escamilla M. Lanzagorta N. Tovilla-Zarate C. A.. (2020). Copy number variants in siblings of Mexican origin concordant for schizophrenia or bipolar disorder. Psychiatry Res. 291:113018. doi: 10.1016/j.psychres.2020.113018 32540681

[ref32] Wahbeh M. H. Avramopoulos D. (2021). Gene-environment interactions in schizophrenia: a literature review. Genes 12:1850. doi: 10.3390/genes12121850 34946799 PMC8702084

[ref33] Walsh T. McClellan J. M. McCarthy S. E. Addington A. M. Pierce S. B. Cooper G. M.. (2008). Rare structural variants disrupt multiple genes in neurodevelopmental pathways in schizophrenia. Science 320, 539–543. doi: 10.1126/science.1155174, PMID: 18369103

[ref34] Wang K. Li M. Hadley D. Liu R. Glessner J. Grant S. F. A.. (2007). PennCNV: an integrated hidden Markov model designed for high-resolution copy number variation detection in whole-genome SNP genotyping data. Genome Res. 17, 1665–1674. doi: 10.1101/gr.6861907, PMID: 17921354 PMC2045149

[ref35] Wu Y. Yao Y.-G. Luo X.-J. (2017). SZDB: a database for schizophrenia genetic research. Schizophr. Bull. 43, 459–471. doi: 10.1093/schbul/sbw102, PMID: 27451428 PMC5605257

[ref36] Xu B. Woodroffe A. Rodriguez-Murillo L. Roos J. L. van Rensburg E. J. Abecasis G. R.. (2009). Elucidating the genetic architecture of familial schizophrenia using rare copy number variant and linkage scans. Proc. Natl. Acad. Sci. U. S. A. 106, 16746–16751. doi: 10.1073/pnas.0908584106, PMID: 19805367 PMC2757863

